# Intensity-Texture Enhanced Swin Fusion for Bacterial Contamination Detection in *Alocasia* Explants

**DOI:** 10.3390/s26072103

**Published:** 2026-03-28

**Authors:** Jiatian Liu, Wenjie Chen, Xiangyang Yu

**Affiliations:** 1 State Key Laboratory of Optoelectronic Materials and Technologies, School of Physics, Sun Yat-sen University, Guangzhou 510275, China; liujt55@mail2.sysu.edu.cn (J.L.); chenwenj5@mail.sysu.edu.cn (W.C.); 2 School of Information and Intelligent Engineering, Guangzhou Xinhua University, 248 Yanjiangxi Road, Machong Town, Dongguan 523133, China; 3 Nanchang Research Institute, Sun Yat-sen University, Nanchang 330096, China

**Keywords:** multispectral image, plant tissue culture, Swin Transformer, image fusion, deep learning, bacterial contamination

## Abstract

**Highlights:**

**What are the main findings?**

- Establishes a multispectral imaging system and dataset for non-destructive *Alocasia* contamination detection.
- Achieves a mean Average Precision (mAP50) of 0.949 using the novel Intensity-Texture enhanced Swin Fusion (ITSF) model.

**What are the implications of the main findings?**

- Introduces a novel, industrially scalable framework for automated contamination detection, advancing the integration of artificial intelligence into plant tissue culture workflows.
- Enables early and accurate identification of bacterial threats, which reduces economic losses, improves production consistency, and supports the sustainable scaling of micropropagation operations.

**Abstract:**

Non-destructive and automated detection of bacterial contamination is a critical prerequisite for ensuring high efficiency production and quality control in plant tissue culture. In this study, we developed a multispectral image acquisition system for *Alocasia* explants and proposed a novel image fusion model, termed Intensity-Texture enhanced Swin Fusion (ITSF). The ITSF framework employs convolutional neural networks to extract texture and intensity features from visible and near-infrared channels. Subsequently, a Swin Transformer-based module is integrated to model long-range spatial dependencies, ensuring cross-domain integration between the texture and intensity features. We formulated a composite loss function to guide the fusion process toward optimal results. This objective function integrates texture loss, entropy weighted structural similarity index (SSIM) and intensity aware dynamic gain guided loss. Experimental results demonstrate that the proposed method significantly enhances the visual saliency of bacteria and achieves superior quantitative performance across a comprehensive range of objective image fusion metrics. The detection performance reached a mean Average Precision (mAP50) of 0.949 with the fused images, satisfying industrial requirements for high-precision inspection, which provides a critical technical solution for the industrialization of automated micropropagation.

## 1. Introduction

The microbial contamination is the primary cause of the explant mortality and the increased production costs in the plant tissue culture [1,2,3]. The microbial contamination in the plant tissue culture originates from the diverse vectors including the non-sterile culture media, the improperly disinfected laboratory apparatus, and the airborne microorganisms [4]. Furthermore, the endophytic microorganisms residing within the internal plant tissues often evade the standard surface sterilization protocols [5]. The contaminants significantly compromise the morphogenic capacity by inhibiting the callus induction and the adventitious shoot proliferation [6,7,8], ultimately leading to the tissue necrosis and the mortality that severely disrupt the production cycles [9,10].

The microbial infections are categorized into the fungal and the bacterial types based on the distinct morphological characteristics [11]. While the fungal contamination is easily identified by the conspicuous mycelia, the bacterial contaminants typically manifest as the translucent biofilms that are nearly indistinguishable during the manual screening [4,12]. This invisibility makes early-stage identification a critical challenge in industrial practice. The conventional methods, such as the manual inspection and the biochemical assays, are limited by the high labor intensity and the exorbitant costs [13]. Consequently, there is an urgent requirement for automated monitoring to enhance the visual saliency of the bacteria and ensure reliable quality control in the modern micropropagation.

Spectral imaging is an advanced imaging technique that integrates optical spectral analysis with image processing technology. By acquiring spectral information across different wavelength ranges and representing its spatial distribution within an image, it enables high-resolution, high-sensitivity detection and analysis of objects. Spectral imaging has emerged as a robust non-destructive technique, widely adopted across agriculture, forensics, and food safety for the detection of adulteration as well as microbial, chemical, and physical contamination [14]. The effectiveness of spectral imaging for bacterial surveillance has been extensively validated across diverse biological contexts. Michael et al. [15] employed hyperspectral imaging to successfully classify and detect several bacterial strains, including *Brucella*, *Salmonella*, and *Escherichia coli*. In another investigation, Jun et al. [16] demonstrated the utility of visible and near-infrared hyperspectral imaging for the quantitative mapping of *E. coli* contamination during fish spoilage. Separately, Soni et al. introduced a deep learning-based framework that combined 1D convolutional neural networks with random forests to distinguish between viable and non-viable spores of *Bacillus cereus* [17]. These collective advancements confirm the diagnostic potential of multispectral imaging as a viable pathway for the rapid, real-time, and non-destructive inspection of plant tissue culture. Utilizing multi-band information through advanced feature extraction offers a reliable approach for the high-precision identification and localized mapping of bacterial contaminants.

While spectral imaging offers substantial potential, the effective extraction of discriminative features from high-dimensional data remains a significant challenge for real-time monitoring. Driven by advancements in computer vision, image fusion has emerged as a practical approach to overcome the representational limitations of single-sensor or single-setting imaging [18]. By combining complementary spatial details and modal attributes from multiple sources, image fusion generates synthesized imagery with increased information density and enhanced scene representation [19], which is vital for downstream tasks such as object detection [20,21]. In micropropagation, the bacterial contaminants are frequently obscured by both the complex textures of the explant surfaces and the cluttered background of the culture medium, which makes it challenging to differentiate the targets within a single spectral band. While the image fusion of the visible texture and the near-infrared sensitivity aim to augment the visual saliency, the existing algorithms fail to sufficiently mine the unique and the correlation information between the individual spectral band. Consequently, the fused results often lack the structural clarity and the intensity contrast required for the reliable automated detection in the complex biological scenarios. This insufficient feature exploration necessitates a more robust framework capable of systematically characterizing the image representation of the bacteria. Crucially, there is currently a total absence of research addressing the automated object detection of bacterial contamination within plant tissue culture environments. This unexplored domain represents a significant technological void, and bridging this gap provides a potent framework for the automated screening of contaminated samples, carrying substantial industrial and economic implications.

The main contributions of this work are summarized as follows:

1. We developed a customized multispectral image acquisition system specifically tailored for *Alocasia* explants and constructed a novel multispectral dataset for bacterial contamination detection. The system facilitates the synchronized acquisition of Red (R), Green (G), Blue (B), and Infrared (IR) channels, establishing a critical database for the automated identification of bacterial contaminants in plant tissue culture;
2. A novel ITSF framework is proposed to facilitate the effective integration of multi-band features. This architecture employs a dual-branch encoder to independently extract texture and intensity features from visible and IR channels. By integrating the Swin Transformer-based modules, the framework models the long-range spatial dependencies and the cross-domain interactions, facilitating the deep fusion of the global and the local feature information;
3. A multi-criteria composite loss function was formulated to optimize the fusion process, incorporating texture loss, entropy weighted SSIM loss, and intensity aware dynamic gain guided loss. This design significantly augments the visual saliency of bacteria while effectively suppressing complex background interference from explant surfaces.

## 2. Related Work

### 2.1. Traditional Image Fusion Methods

Traditional image fusion methodologies primarily comprise Principal Component Analysis (PCA), wavelet transforms, saliency-based fusion, and sparse representation [19]. For instance, Li et al. [22] integrated PCA with Morphology-Hat transforms to process the low-frequency components of infrared and visible images, effectively preserving critical luminance information. In another study, Chai et al. [23] utilized the Quaternion Wavelet Transform (QWT) coupled with multi-feature integration, demonstrating robust efficacy across diverse image fusion scenarios. Furthermore, Liu et al. [24] introduced a framework based on Convolutional Sparse Representation (CSR), where source images are decomposed into base and detail layers to facilitate both multi-focus and multimodal fusion tasks. Additionally, Zhao et al. [25] proposed a visible and infrared image fusion method centered on multi-window visual saliency extraction, which enhances fusion performance by calculating localized saliency maps across various window scales.

While these traditional approaches have achieved a degree of success, they rely heavily on hand-crafted fusion rules designed from prior knowledge of specific scenarios. Such manually defined rules often struggle to adapt to complex and dynamic environments, leading to various adverse effects. These include significant spectral or spatial distortion and a frequent failure to highlight the distinct features of target objects [26].

### 2.2. Deep Learning-Based Methods for Image Fusion

In recent years, deep learning-based image fusion has gained significant traction due to its robust feature extraction capabilities and superior generalization across diverse environments. Current frameworks are primarily categorized into those based on Convolutional Neural Networks (CNNs), Generative Adversarial Networks (GANs), Transformers, and Diffusion models. For instance, Liu and Wu introduced DenseFuse [27], a framework comprising an encoder–decoder architecture where the encoder leverages convolutional layers and dense blocks to preserve multi-scale features, followed by a fusion strategy and a multi-layer CNN-based decoder for image reconstruction. Ma et al. [28] pioneered the application of GANs to infrared and visible image fusion with FusionGAN. This approach establishes adversarial training between a generator and a discriminator to yield fused results that encapsulate the most salient information from source images. However, these convolution-based models are intrinsically limited by the local receptive field of convolutional kernels, which hinders their ability to represent long-range dependencies and often results in the loss of critical global contextual information.

To address these limitations, the Transformer architecture has been introduced to capture global dependencies. Originally proposed by Vaswani et al. [29] for machine translation, this technology enables the direct modeling of global context. In the computer vision domain, Dosovitskiy et al. introduced the Vision Transformer (ViT) [30], which processes images by reshaping them into sequences of flattened 2D patches. Subsequently, Liu et al. [31] proposed the Swin Transformer to enhance efficiency in tasks like image classification and dense prediction. By constraining self-attention computation within localized windows and introducing shifted window connections, the Swin Transformer achieves a hierarchical representation with significantly reduced computational complexity.

These Transformer architectures are employed in image fusion. VS et al. [32] first integrated the Transformer architecture through the IFT model, employing a two-stage training strategy to extract and fuse features via an auto-encoder and a spatial Transformer. Wang et al. [33] further developed a residual Swin Transformer-based fusion network, incorporating dedicated stages for global feature extraction and reconstruction. Moreover, Ma et al. [34] proposed a hybrid architecture that utilizes CNNs for shallow local feature extraction and Swin Transformers for deep global feature modeling. Their approach employs an attention-guided cross-domain fusion module to integrate intra-domain and inter-domain interactions, achieving state-of-the-art performance across multiple fusion tasks.

Recently, image fusion frameworks based on Diffusion models [35] have also emerged. Zhao et al. [36] introduced Denoising Diffusion image Fusion Model (DDFM), which generates high-quality fused imagery without requiring fine-tuning of the pre-trained model. Liang et al. [37] proposed FusionINV, which inverts infrared images into a noise feature space and injects visible information through the inverted features of the visible modality. Despite their impressive performance, diffusion-based frameworks are characterized by prolonged inference times and heavy computational requirements [38], rendering them less suitable for the real-time constraints of industrial-grade inspection.

## 3. Materials and Methods

### 3.1. Sample Preparation

The plant materials utilized in this study were *Alocasia* explants, sourced from a commercial micropropagation facility in Conghua District, Guangzhou, Guangdong Province. As a prominent genus within the Araceae family, *Alocasia* possesses substantial ornamental and medicinal value [39] and is frequently propagated via industrial-scale tissue culture, making it a highly representative model for contamination research. For data collection, 20 culture vessels were strategically selected, comprising 16 bottles with bacterial contamination and 4 uncontaminated bottles. This sampling strategy was implemented to ensure a balanced representation across the different sample categories within the study.

### 3.2. Data Acquisition and Preprocessing

**Figure 1:**
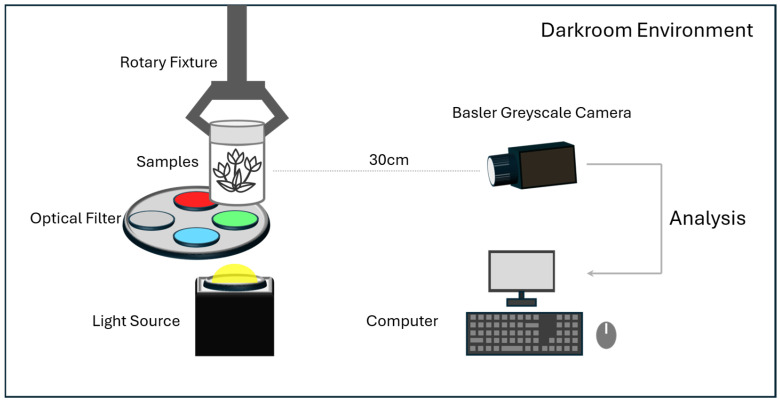
Schematic representation of the multispectral image acquisition system architecture.

A custom-built multispectral image acquisition system was developed for this study, the architecture of which is illustrated in Figure 1. The core imaging component is a Basler monochrome camera (acA5472-5gm, Basler, Ahrensburg, Germany), providing a high resolution of 5472×3648 pixels with a field of view (FOV) of 18.5° × 15.5°. Illumination is provided by a wide-spectrum, constant-temperature light source (GX-IRSV-FL-4095, Guangzhou Guangxin Technology Co., Ltd., Guangzhou, China), covering a spectral range of 400–950 nm.

During the acquisition process, multispectral data are captured across four discrete bands by rotating a motorized filter wheel. The specific wavelength ranges for these channels are defined as follows: Blue (430–470 nm), Green (535–575 nm), Red (640–680 nm), and Infrared (820–880 nm). To ensure comprehensive spatial coverage, each sample bottle was positioned on a rotating fixture and imaged at 30° intervals. At each angular position, the four spectral channels were captured sequentially by rotating the motorized filter wheel, ensuring precise spatial alignment across all bands.

After removing anomalous or low-quality frames, the final dataset comprised 222 sets of multispectral images (each set containing R, G, B, and IR bands). This includes 192 sets of contaminated samples and 30 sets of uncontaminated (blank) samples. All captured images underwent preliminary preprocessing, including localized cropping and denoising, to preserve high-quality image information and ensure data consistency for subsequent model training.

### 3.3. Methodology

#### 3.3.1. Overall Architecture

**Figure 2:**
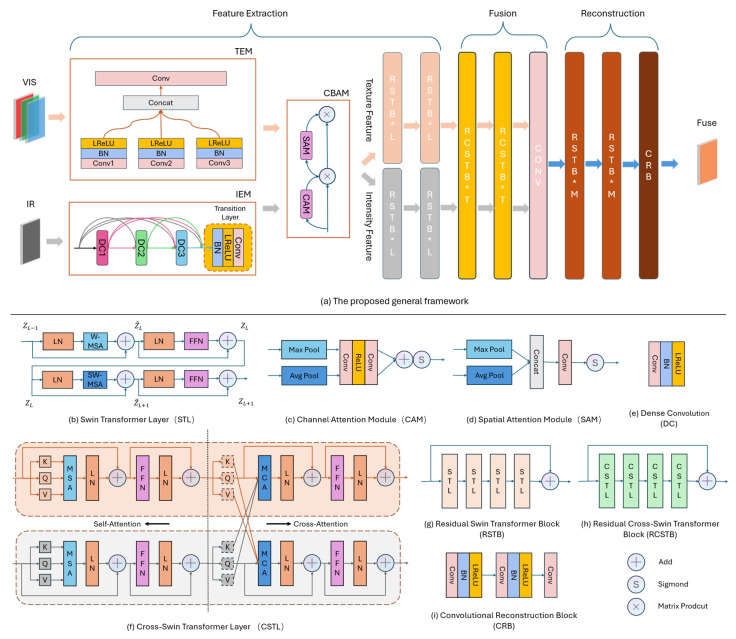
The overview of the proposed ITSF framework, which takes the visible images $I_{VIS}$ and the infrared images $I_{IR}$ as the inputs and outputs the fused image. The architecture consists of Feature Extraction, Fusion, and Reconstruction. The detailed architecture of the proposed fusion blocks. The notation ‘*’ denotes the architectural depth of the parent modules (e.g., RSTB and CRSTB), specifying the number of successive Swin Transformer Layers (STL) cascaded within each block.

Figure 2a illustrates the overall architecture of the proposed ITSF model. The framework processes two primary inputs: a visible light image $I_{VIS}\in R^{H\times W\times C_{1}}$ and an infrared image $I_{IR}\in R^{H\times W\times C_{2}}$. Specifically, $I_{VIS}$ is constructed by concatenating the Red, Green, and Blue spectral channels along the channel dimension with equal weighting, ensuring a balanced contribution of visible spectral bands. The model’s objective is to produce a synthesized fused image $I_{F}\in R^{H\times W\times C_{out}}$, where H and W denote the height and width of the imagery, respectively, while $C_{1}$, $C_{2}$, and $C_{out}$ designate the channel counts for the visible light input, the infrared input, and the resulting fused output. The model’s workflow is organized into the following functional components:

Feature Extraction: The feature extraction process is designed to capture the multidimensional attributes of the bacterial contaminants. The visible image $I_{VIS}$ is processed by the Texture Extraction Module (TEM) to capture initial textural representations, focusing on the fine-grained morphological details such as the mucilaginous filaments and the turbid boundaries that characterize the bacterial biofilms. Simultaneously, the infrared image $I_{IR}$ passes through the Intensity Extraction Module (IEM) to process initial intensity information, enhancing the contrast between the translucent bacteria and the complex culture medium. These features are subsequently refined by the Convolutional Block Attention Module (CBAM) [40], which adaptively modulates the feature maps through sequential spatial and channel attention mechanisms to preserve key information. To bridge the gap between local representations and global context, the data then enters Residual Swin Transformer Blocks (RSTBs) to capture long-range dependencies, yielding two distinct representations: the texture feature domain and the intensity feature domain.

Fusion: Following the extraction of two features domains enriched with global semantic information, the Residual Cross-Swin Transformer Block (RCSTB) is employed to further mine and aggregate both the intra- and inter-domain global context. These interacted features are subsequently consolidated into a unified fused representation through a convolutional layer, effectively synthesizing the latent information into a fused feature output.

Reconstruction: The fused feature is first passed through RSTBs for global information restoration, ensuring the structural integrity of the scene. This is followed by a Convolutional Reconstruction Block (CRB) designed for fine-grained local detail refinement. This hierarchical process ultimately generates a synthesized fusion image.

#### 3.3.2. Feature Extraction

To fully leverage the complementary information from the visible and infrared spectra, we implemented distinct feature extraction protocols tailored to each modality. For the visible light modality, a multi-scale convolutional strategy is employed to capture rich textural details across different spatial frequencies. Specifically, three parallel convolutional layers, Conv1, Conv2, and Conv3, are utilized with kernel sizes of 3×3, 5×5, and 7×7, respectively. Each layer follows a consistent architectural template, designated as $H_{CBL}\left(\cdot \right)$, which sequentially integrates a convolutional operation, a Batch Normalization (BN) layer, and a Leaky Rectified Linear Unit (LReLU) activation function. The mapping functions corresponding to these multi-scale kernels are denoted as $H_{CBL1}\left(\cdot \right)$, $H_{CBL2}\left(\cdot \right)$, and $H_{CBL3}\left(\cdot \right)$. The resulting feature maps from these three branches are concatenated along the channel dimension and further integrated through a fusion convolution layer $H_{Conv}\left(\cdot \right)$, with a 3×3 kernel to generate the preliminary texture information.

Regarding the IR modality, the DenseBlock architecture is adopted for the deep extraction of intensity information, represented by the function $H_{DB}\left(\cdot \right)$. By leveraging dense connectivity, this module facilitates feature reuse across layers and mitigates the loss of low-level information during deep feature abstraction. This ensures the effective propagation of crucial intensity attributes and structural cues, which are vital for maintaining the clarity of the fused imagery. As illustrated in Figure 2g, this module consists of three consecutive Dense Convolutional (DC) layers [41] with 3×3 kernels. Subsequently, a Transition Layer is employed to compress the feature maps and mitigate model complexity, yielding the preliminary intensity information.

To further highlight salient diagnostic features and suppress background noise, the CBAM [40], represented by the function $H_{CBAM}\left(\cdot \right)$, sequentially integrates a CAM and a SAM. As illustrated in Figure 2c,d, this mechanism adaptively modulates the texture and intensity feature maps across both spatial and channel dimensions. This dual-attention refinement ensures that the model focuses on the most informative regions of the multispectral data. Consequently, the feature extraction and refinement process is mathematically formulated as follows:

(1) $$I_{TEX}=H_{CBAM}\left(H_{Conv}\left(concat\left(H_{CBL1}\left(I_{VIS}\right),\;H_{CBL2}\left(I_{VIS}\right),H_{CBL3}\left(I_{VIS}\right)\right)\right)\right),$$

(2) $$I_{INT}=H_{CBAM}(H_{DB}{(I}_{IR})).$$

Nevertheless, while CNN-based operations excel at capturing local feature details, the modeling of global dependencies is indispensable for achieving a rational distribution of global image luminance [34]. The shifted window mechanism of the Swin Transformer facilitates cross-window information interaction, allowing the model to capture global dependencies while significantly reducing computational complexity. As illustrated in Figure 2b, Swin Transformer Layer (STL) typically consists of an even number of layers forming consecutive pairs [31]. The odd-numbered layers perform self-attention within localized windows, while the even-numbered layers implement a window shifting operation to enable information exchange across newly formed window boundaries. During the computation process, an input feature map of size H×W is first partitioned into $\frac{H\times W}{N^{2}}$ non-overlapping windows, where each window possesses a spatial dimension of N×N. Assuming the input for a single window in an odd-numbered layer is denoted as $Z_{L-1}\in R^{N^{2}\times ∁}$, where ∁ represents the assigned channel count, the self-attention computation is formulated as follows:

(3) $$\left\{Q,K,V\right\}=\{Z_{L-1}W^{Q},Z_{L-1}W^{K},Z_{L-1}W^{V}\},\;{\tilde{Z}}_{L}=MSA(LN\left(Q,K,V\right))+Q,\;Z_{L}=FFN\left(LN({\tilde{Z}}_{L})\right)+{\tilde{Z}}_{L},$$

where Q,K,V denote the query, key, and value matrices, respectively, while $W^{Q}$, $W^{K}$, $W^{V}$ $\in R^{C\times ∁}$ represent the learnable weight matrices shared across different windows. Following the LN operation, MSA enables the model to incorporate diverse attention distributions and focus on information from various perspectives. In our study, the number of heads h is set to 4 to facilitate the parallel execution of the attention function h times. The attention function is defined as Equation (4).

(4) $$Attention\left(Q,K,V\right)=softmax\left(\frac{QK^{T}}{\sqrt{d_{k}}}+B\right)V,$$

where $d_{k}$ represents the dimension of the keys, and B denotes the learnable relative position bias. Subsequently, a feedforward neural network (FFN) consisting of two Multi-Layer Perceptron (MLP) layers with Gaussian Error Linear Unit (GELU) activation is deployed to refine the feature tokens generated by MSA. Notably, the LN operation is applied prior to both the MSA and the FFN to better accommodate variations in scale and discrepancies across different image data. Furthermore, both components incorporate residual connections to facilitate stable gradient flow. The computation for the FFN can be formulated as follows:

(5) $$FFN\left(X\right)=GELU\left(W_{1}X+b_{1}\right)W_{2}+b_{2}.$$

**Figure 3:**
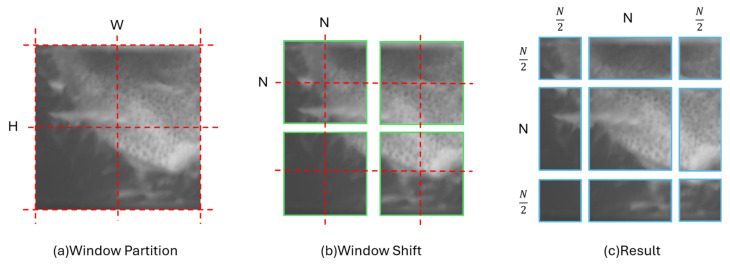
The schematic illustration of the shifted window mechanism. The figure is divided into three primary components: (**a**) the initial window partitioning, (**b**) the schematic representation of the window shifting process, and (**c**) the final partitioning configuration after the displacement.

In Equation (5), GELU represents the Gaussian Error Linear Unit. The operations described above pertain to the odd-numbered layers of the STL. Conversely, the even-numbered layers involve a shifted window operation to facilitate cross-window connectivity. This shifted window mechanism entails displacing the original window partitions by $\left(\left\lfloor \left.\frac{N}{2}\right\rfloor \right.,\left\lfloor \left.\frac{N}{2}\right\rfloor \right.\right)$ pixels relative to the initial configuration, thereby generating a new set of windows. As illustrated in Figure 3, this alternating scheme between standard and shifted window operations across successive layers provides the interaction between information residing in disparate windows within the STL.

The two previously obtained information, $I_{TEX}$ and $I_{INT}$, are processed by the ${RSTB}_{L}$, which comprises L STLs equipped with residual connections. We utilize the $N_{ext}$ ${RSTB}_{L}s$ during the feature extraction stage, which effectively integrates a synergy of rich local details and global dependencies. This operation is represented by the function $\Phi_{{RSTB}_{L}}^{N_{ext}}\left(\cdot \right)$, which generates two enhanced feature domains, $F_{TEX}$ and $F_{INT}$. The corresponding formulations are as follows:

$$F_{TEX}=\Phi_{{RSTB}_{L}}^{N_{ext}}\left(I_{TEX}\right),$$

(6) $$F_{INT}=\Phi_{{RSTB}_{L}}^{N_{ext}}\left(I_{INT}\right).$$

#### 3.3.3. Fusion

During the fusion stage, inspired by the attention-guided cross-domain fusion mechanism proposed in SwinFusion [34], we incorporate the Cross-Swin Transformer Layer (CSTL), which integrates both self-attention and cross-attention computations. In the self-attention phase, the texture and intensity feature domains, $F_{TEX}$ and $F_{INT}$, undergo independent attention processing within their respective domains. This procedure is analogous to the operations within STL detailed in the feature extraction section. Conversely, the cross-attention phase utilizes MCA rather than MSA to aggregate global contextual information across disparate modalities, as illustrated in Figure 2f. For the feature modalities $F_{TEX}$ and $F_{INT}$, the specific cross-attention calculation is defined as follows:

(7) $$\{Q_{1},K_{1},V_{1}\}=\{F_{TEX}W^{Q_{1}},F_{TEX}W^{K_{1}},F_{TEX}W^{V_{1}}\},\;\{Q_{2},K_{2},V_{2}\}=\{F_{INT}W^{Q_{2}},F_{INT}W^{K_{2}},F_{INT}W^{V_{2}}\},\;{\tilde{Z}}_{1}=LN\left(MCA(Q_{1},K_{2},V_{2})\right)+Q_{1},\;{\tilde{Z}}_{2}=LN\left(MCA(Q_{2},K_{1},V_{1})\right)+Q_{2},\;Z_{1}=LN\left(FFN({\tilde{Z}}_{1})\right)+{\tilde{Z}}_{1},\;Z_{2}=LN\left(FFN({\tilde{Z}}_{2})\right)+{\tilde{Z}}_{2}.$$

For the query $Q_{1}$ derived from $F_{TEX}$, MCA integrates cross-domain information by utilizing the key $K_{2}$ and value $V_{2}$ generated from $F_{INT}$, while a residual connection is employed to preserve the original information from $F_{TEX}$. A symmetrical operation is concurrently performed for $F_{INT}$. Our model incorporates the ${RCSTB}_{L}$, which consists of T CSTLs and is reinforced by a residual connection that bypasses the entire block. Each CSTL comprises self-attention and cross-attention fusion units, meticulously designed to alternately integrate global intra-domain and inter-domain interactions. The $N_{fus}$ ${RCSTB}_{L}s$ is denoted by the function $\Psi_{{RCSTB}_{L}}^{N_{fus}}\left(\cdot \right)$, which culminates in the generation of the global deep features, ${F^{\prime }}_{TEX}$ and ${F^{\prime }}_{INT}$. The corresponding mathematical expressions are as follows:

(8) $${F^{\prime }}_{TEX}=\Psi_{{RCSTB}_{L}}^{N_{fus}}\left(F_{TEX}\right),\;{F^{\prime }}_{INT}=\Psi_{{RCSTB}_{L}}^{N_{fus}}\left(F_{INT}\right).$$

In the subsequent step, ${F^{\prime }}_{TEX}$ and ${F^{\prime }}_{INT}$ are integrated through a channel concatenation operation and processed by the Conv layer. This process exploits the intrinsic weight sharing property of convolutional kernels to ensure that feature representations across disparate spatial coordinates are processed with strict parameter consistency, thereby strengthening the positional robustness of the fusion framework. This procedure can be expressed as

(9) $$D_{f}=H_{Conv}\left(Concat({F^{\prime }}_{TEX},{F^{\prime }}_{INT})\right),$$

where $D_{f}$ represents the deep feature representation characterized by global contextual information, which is derived from the fusion of the texture and intensity modalities.

#### 3.3.4. Reconstruction

Following the fusion of complementary attributes across the intensity and texture feature domains, the aggregated deep features enriched with global semantic information undergo a progressive restoration process. Specifically, the ${RSTB}_{M}$ comprising M STLs along with a residual connection is utilized to reconstruct the latent feature maps from a global perspective. Subsequently, a meticulously designed CNN network is employed to restore the original image details from feature maps. The reconstruction process is defined as follows:

(10) $$S_{f}=H_{CRB}\left(\Gamma_{{RSTB}_{M}}^{N_{rec}}\left(D_{f}\right)\right),$$

where $\Gamma_{{RSTB}_{M}}^{N_{rec}}\left(\cdot \right)$ denotes the operation of the $N_{rec}$ ${RSTB}_{M}s$, and $H_{CRB}\left(\cdot \right)$ signifies the CNN-based reconstruction module. As illustrated in Figure 2i, the architecture of CRB comprises two convolutional layers, each integrated with a BN layer and a LReLU activation function. Subsequently, a final convolutional layer is appended to reconstruct the spatial dimensions and restore the image information.

### 3.4. Loss Function

To effectively highlight the bacterial characteristics within tissue culture vessels, a specialized compound loss function is meticulously formulated. In these environments, bacteria typically manifest as floating flocculent and translucent entities, possessing intricate texture details but maintaining low luminance due to their semi-transparent nature. We identify two primary forms of background interference: (i) Fog-like blurring within the culture medium caused by light scattering, characterized by diminished brightness and textural detail. (ii) The localized overexposure within the specific regions, resulting from the non-uniform distribution of the medium or the specular reflections originating from the container surfaces, which masks the underlying spatial distribution of the bacteria. To enhance the target features while suppressing these artifacts, the proposed objective function integrates the intensity aware dynamic gain guided loss, the texture loss, and the entropy weighted SSIM loss. The total loss $L_{TOTAL}$ is defined as follows:

(11) $$L_{TOTAL}=\lambda_{1}L_{IDG}+\lambda_{2}L_{TEXT}+\lambda_{3}L_{EW-SSIM},$$

where $\lambda_{1}$, $\lambda_{2}$, $\lambda_{3}$ denote the respective weighting coefficients.

#### 3.4.1. Entropy Weighted SSIM Loss

SSIM is a widely utilized metric that evaluates image distortion through three primary components: luminance, contrast, and structure [42]. Typically, the SSIM loss is employed to constrain the structural consistency between the fused image $I_{F}$ and the input sources $I_{VIS}$ and $I_{IR}$. To ensure that the fused result incorporates more comprehensive information, we adopt an entropy weighted SSIM method. This approach enables the model to maximize information extraction from four distinct sources: $I_{R}$, $I_{G}$, $I_{B}$ and $I_{IR}$. Specifically, the entropy weighted SSIM loss ($L_{EW-SSIM}$) is mathematically formulated as

(12) $$L_{EW-SSIM}=\sum_{X\in \left\{R,G,B,IR\right\}}1-w_{X}\cdot SSIM\left(X,F\right),$$

where X denote the source image and F represent the fused image. SSIM is formulated as follows:

(13) $$SSIM\left(X,F\right)=\frac{(2\mu_{X}\mu_{F}+c_{1})\left(2\sigma_{XF}+c_{2}\right)}{\left({\mu_{X}}^{2}+{\mu_{F}}^{2}+c_{1})({\sigma_{X}}^{2}+{\sigma_{F}}^{2}+c_{2}\right)},$$

where $\mu_{X}$ and $\mu_{F}$ denote the local mean values of the images X and F, respectively. $\sigma_{X}$ and $\sigma_{F}$ represent the standard deviations of X and F, while $\sigma_{XF}$ signifies the covariance between the two images. Finally, $c_{1}$ and $c_{2}$ are constants incorporated to maintain numerical stability.

In Equation (12), the normalized weight $w_{X}$, which adaptively balances the contribution of each modality based on its entropy, is defined as follows:

(14) $$w_{X}=\frac{\kappa_{X}}{\sum_{Y\in \left\{R,G,B,IR\right\}}\kappa_{Y}},$$

where global average entropy $\kappa_{X}$ is calculated in a uniform manner for all four source images, which can be formulated as follows:

(15) $$\kappa_{X}=\frac{1}{HW}\sum_{i=1}^{H}\sum_{j=1}^{W}E_{X}\left(i,j\right).$$

In Equation (15), $E_{X}\left(i,j\right)$ signifies the local information entropy. For the k×k neighborhood $\Omega_{ij}$ centered at the spatial position $\left(i,j\right)$, the calculation is formulated as follows:

(16) $$E_{X}\left(i,j\right)=-\sum_{b=0}^{N-1}P_{\Omega_{ij}}\left(b\right)\cdot {log}_{2}P_{\Omega_{ij}}\left(b\right).$$

In Equation (16), $P_{\Omega_{ij}}\left(b\right)$ represents the probability of the b−th gray level occurring within the local neighborhood $\Omega_{ij}$. This metric effectively quantifies the regional textural complexity within the feature domains.

#### 3.4.2. Texture Loss

A primary objective of image fusion is to consolidate the texture details from the source images into a single unified representation. We observe that the texture details within the source images can be effectively aggregated through a maximum gradient selection strategy. Consequently, the texture loss, denoted as $L_{TEXT}$, is designed to supervise the network in preserving the maximum possible level of textural information. The formulation is as follows:

(17) $$L_{TEXT}={\frac{1}{HW}∥\left|\nabla I_{f}\right|-\max_{X\in \left\{R,G,B,\mathrm{IR}\right\}}\left|\nabla I_{X}\right|\;∥}_{1}$$

In this formulation, ∇ denotes the Sobel gradient operator, which is employed to measure the textural information of the images. The symbol |⋅| signifies the absolute operation, while ‖⋅‖_1_ represents the $L_{1}$ norm. Furthermore, $max\left(\cdot \right)$ refers to the pixel level maximum selection.

#### 3.4.3. Intensity Aware Dynamic Gain Guided Loss

A high-quality image fusion algorithm should generate a fused image with appropriate intensity, dynamically adapted to the global appearance information of the source images. Therefore, we propose the intensity aware dynamic gain guided loss, denoted as $L_{IDG}$, to supervise the network in capturing and preserving the desired intensity information from the source images. The formulation is as follows:

(18) $$L_{IDG}=\;\begin{array}{c} E\left[\left|I_{fused}-\left(\begin{array}{c} {\hat{m}}_{bright}\cdot \sum_{X\in \left\{R,G,B,IR\right\}}Softmax\left(\frac{\nabla I_{X}}{T}\right)\cdot I_{X}\\ +{\hat{m}}_{dark}\cdot \sum_{X\in \left\{R,G,B,IR\right\}}I\left(X={argmax}_{Y\in \left\{R,G,B,IR\right\}}\nabla_{Y}^{norm}\right)\cdot g_{X}\cdot I_{X} \end{array}\right)\right|\right]. \end{array}$$

In Equation (18), ${\hat{m}}_{bright}$ denotes the bright mask, while the dark mask ${\hat{m}}_{dark}$ represents its complement. The mathematical expressions for the masks are presented in Equation (19). Within this formulation, $\tau_{0}$ is set to 0.3, and I(⋅) signifies the indicator function, which yields a value of 1 when the specific condition is satisfied and 0 otherwise.

(19) $$\left\{\begin{array}{c} {\hat{m}}_{bright}=I\left(I_{fused}\geq \tau_{0}\cdot maxI_{fused}\right)\\ {\hat{m}}_{dark}=I\left(I_{fused}<\tau_{0}\cdot maxI_{fused}\right) \end{array}\right..$$

Within the bright mask region, a gradient weighting method is utilized to assess the high intensity areas of the source modalities. If a source image exhibits a more prominent gradient within the bright mask, the fused image is biased toward preserving the information from that source image.

This strategy effectively prevents the model from retaining the overexposed and blurry artifacts that may be present on one channel, while ensuring that the effective low-brightness information from other source images is not neglected. In our framework, the gradient weights of the source images are implemented via the $Softmax\left(\cdot \right)$, as formulated in Equations (18) and (20). In Equation (18), $T=sqrt\left(1/\xi \right)$, ξ denotes the total number of source images, ensuring a reasonable distribution of image brightness.

(20) $$Softmax\left(x_{i}\right)=\frac{e^{x_{i}}}{\sum_{j\in \left\{R,G,B,IR\right\}}e^{x_{j}}},$$

Regarding the dark mask regions defined by ${\hat{m}}_{dark}$, the gradient weights serve as the primary reference for selection. Specifically, the model identifies and selects the pixels possessing the richest textural information from the four modalities to serve as the reference for the fused image. Furthermore, a gradient-based dynamic gain is introduced to highlight the luminance of these regions. The dynamic gain $g_{X}$, which is calculated based on the average gradient of the source images, operates within the interval of [1.0, 1.25]. Here, $g_{min}$ and $g_{max}$ are set to 1.0 and 1.25 respectively.

(21) $$g_{X}=g_{min}+\frac{{\bar{\nabla }}_{X}-min{\bar{\nabla }}_{Y}}{max{\bar{\nabla }}_{Y}-min{\bar{\nabla }}_{Y}}\cdot \left(g_{max}-g_{min}\right).$$

## 4. Experiments

In this section, we evaluate the effectiveness and generalization of our proposed model within the collected tissue culture data fusion scenarios through both quantitative and qualitative comparisons. We first present the experimental configuration and implementation details. Subsequently, we compare our method ITSF with several mainstream image fusion algorithms to demonstrate its superiority. Finally, we conduct a series of ablation studies to investigate the specific contributions of the proposed model architecture and the loss functions.

### 4.1. Experimental Configuration and Implementation Details

For the dataset utilized in this study, the training, validation, and test sets are partitioned in a ratio of 8:1:1. To evaluate generalization, the test set was specifically designed to include all images from two independent vessels that were entirely excluded from the training process. For other samples in the test sets, independence was maintained by using unique 30° rotational viewpoints that did not overlap with the training set. This ensures that the model identifies bacterial contamination based on generalized features rather than vessel-specific backgrounds. To mitigate model overfitting, we implement several data augmentation techniques on the training data, including image rotation, flipping, and random cropping, with corresponding probabilities of 0.3, 0.3, and 0.1, respectively. The input images comprise four modalities (R, G, B, and IR), with a spatial resolution of (200, 90), and are normalized to the range of [0, 1].

The batch size is set to 4, the maximum number of training epochs is 100, and early stopping is triggered after 5 epochs of no improvement. The model parameters are optimized using the AdamW optimizer, with an initial learning rate of 0.0001 and a weight decay coefficient of 0.01. To ensure the reproducibility of the results, all experiments were conducted with a fixed random seed of 42.

Regarding the Swin Transformer configuration, the window size is set to 5, the patch size is designated as 1×1, and the sequence length following patch embedding is 48. $N_{ext}$, $N_{fus}$, and $N_{rec}$ are all set to 2. The number of layers L, T, and M for the STL, the CSTL, and the RSTL modules are all configured to 4. The proposed model is implemented on the PyTorch platform [43]. All experiments are conducted on an NVIDIA TITAN RTX 4090 GPU and a 16 vCPU Intel Xeon Gold 6430 processor. The software environment is built on Python 3.9.12 and PyTorch 2.5.1 with CUDA 12.4 acceleration.

### 4.2. Comparative Methods and Image Evaluation Metrics

To comprehensively evaluate the performance of the proposed ITSF method, we select six representative comparative algorithms encompassing both traditional techniques and deep learning approaches. The traditional methods include PCA and Wavelet Transform, while the deep learning methods consist of DenseFuse [27], FusionGAN [28], SwinFusion [34], and DDFM [36].

To establish a multidimensional evaluation system, six representative objective metrics are employed to quantify the fusion performance across four distinct dimensions. Specifically, the Standard Deviation (SD) [44] is utilized to assess the contrast and visual sharpness of the fused image, while the Sum of Correlation Differences (SCD) [45] evaluates the information integration from the source modalities via the correlation of difference images. To measure the perceptual quality from the perspective of human visual perception, the Visual Information Fidelity (VIF) [46] and the Qabf metric [47] are introduced. Finally, the Peak Signal-to-Noise Ratio (PSNR) and the Feature Mutual Information (FMI) [48] are adopted to provide quantitative analysis of global pixel fidelity and local feature information preservation, respectively. Higher values for SD, SCD, Qabf, VIF, and PSNR signify superior fusion performance and enhanced image quality.

### 4.3. Quantitative Comparison of Image Metrics

To validate the quantitative superiority of our proposed method, we utilize the evaluation set comprising 24 test images and perform a comprehensive assessment across six objective metrics. Table 1 summarizes the average values of these metrics across various fusion methods.

Regarding the SD metric, our method achieves the highest score, indicating that the fused images generated by the proposed model possess the optimal contrast and the highest level of visual clarity. Analysis of the SCD metric reveals that our method significantly outperforms the runner up, the PCA method, by a substantial margin. This suggests that the proposed ITSF effectively exploits the complementary differences between the source images, thereby maximizing the information retention.

In the visual perception dimensions, the proposed method demonstrates a highly competitive performance. Although the model is marginally inferior to the PCA method in terms of the QABF metric, it secures the highest VIF score among all the comparative algorithms. Given the superiority of this method in terms of VIF and its second-best results in Qabf, it may be concluded that this method yields the most favorable assessment in visual perception evaluation.

For the FMI and PSNR metrics, our method ranks as the runner up. However, when considering the overall performance relative to the top ranked methods (PCA and DenseFuse) for these specific metrics, our approach demonstrates the most balanced and superior overall performance across these two metrics.

**Figure 4:**
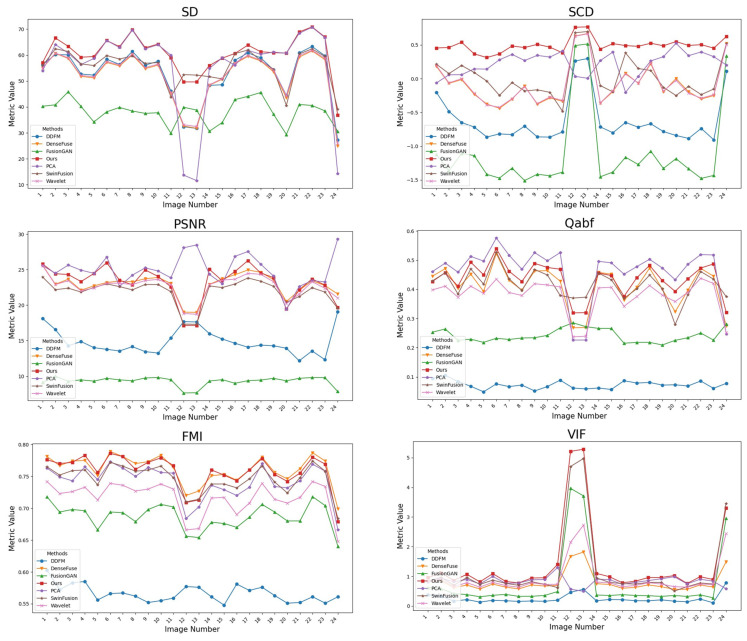
Objective image metrics for different methods on our dataset.

To further illustrate the generalization capability of our method, Figure 4 provides a detailed observation of the metric results across individual images. It is observed that the traditional PCA method exhibits inconsistent performance, specifically showing poor results in the SD and Qabf metrics for images 12 and 13, which indicates a lack of robustness. In contrast, the deep learning methods maintain stable performance across the entire dataset, demonstrating a broader generalization capability. Even when fluctuations occur, such as in the VIF and SCD metrics, they typically manifest as further improvements in the fusion quality.

### 4.4. Qualitative Visual Comparison

To verify that the proposed image fusion network can effectively synthesize the information across the four source modalities and maximize the saliency performance of the bacteria, we conduct the following qualitative visualizations.

**Figure 5:**
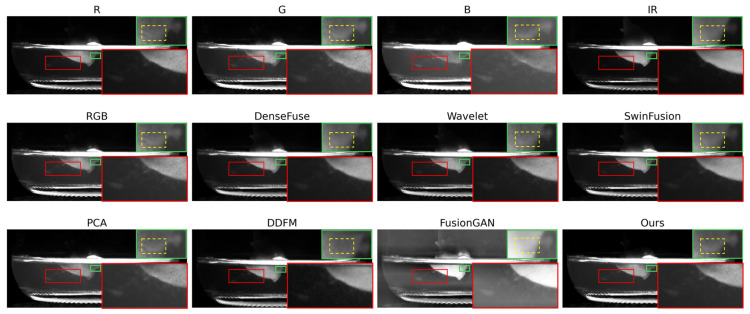
The visual characterization of contaminated tissue culture samples. The red boxes indicate the appearance of the bacteria situated outside the explants within the culture medium. The green boxes represent the distribution of the bacteria on the explant surfaces, while the yellow dashed boxes specifically highlight the detailed morphology of the bacterial contaminants.

As illustrated in Figure 5, a detailed examination of the specific regions further validates the superiority of the proposed ITSF method. In the red boxes, which highlight the bacteria manifested as the floating flocculent substances outside the tissue culture, the original information from the R and B channels provides the clearest representation.

Comparing the various fused results, the PCA method and our proposed method emerge as optimal approaches. These methods not only preserve the textural information of the bacteria to the maximum extent but also significantly suppress the interference caused by the background scattering noise.

Furthermore, the green boxes display the bacteria located on the surface of the tissue culture. While the B channel captures the overall morphology of the bacteria, the G channel effectively reveals the distribution of the bacteria across the surface. In comparative analysis, most fusion algorithms, except for the PCA method and the proposed ITSF, fail to distinguish the bacteria from the tissue culture surface. Our method ensures that the bacterial targets are clearly delineated, maintaining high contrast and structural clarity even within the complex surface textures.

**Figure 6:**
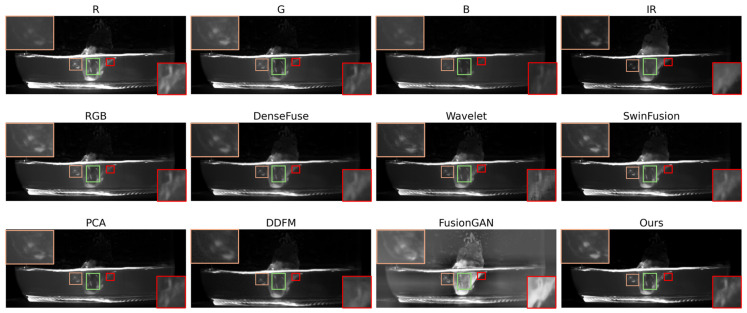
The visual representation of the additional contaminated tissue culture samples. The red and the orange boxes signify the bacterial colonies situated within the culture medium surrounding the explants. The green boxes highlight the bacterial contaminants localized within the internal tissues of the explant surfaces.

As illustrated in Figure 6, the visual advantages of the proposed ITSF method are further demonstrated through a localized analysis of the bacterial features. In the red boxes, the bacteria appear most prominently within the R and G channels. When comparing the various fusion outcomes, the PCA method and our proposed method remain the superior approaches, successfully capturing the critical details that other algorithms overlook.

The green boxes highlight the bacteria distributed across the tissue culture surface, characterized by four bright stripes. These features are most visible on the R and G channels. Among the comparative methods, both the Wavelet method and the proposed ITSF perform remarkably well, significantly enhancing the visual contrast of the bacteria and making the biological structures more distinguishable from the background.

Furthermore, in the orange boxes, the bacteria are most evident in the IR and G channels. Evaluation of the fusion results reveals that the SwinFusion method and our proposed method both deliver outstanding performance. These two approaches effectively suppress the background noise while simultaneously enhancing the luminance and the textural information of the bacteria. Overall, the proposed ITSF consistently maintains high fidelity across all regions, demonstrating its ability to adaptively integrate the most informative features from the source images.

**Figure 7:**
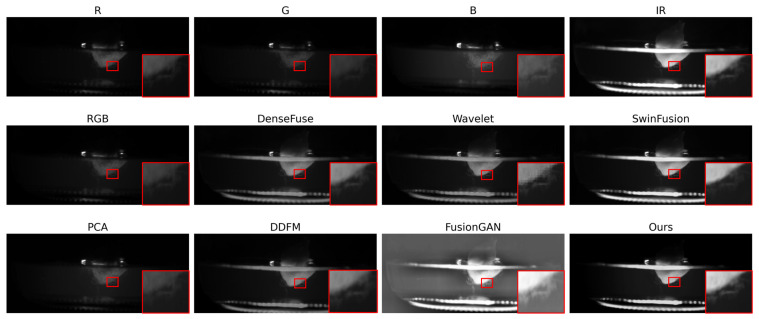
The characterization of the contaminated plant tissue samples exhibiting the low brightness within the visible spectrum. The red region indicates the bacteria surrounding the explants within the culture medium.

As illustrated in Figure 7, we analyze the performance of the fusion methods under the low-luminance conditions within the visible channels. In such scenarios, the bacteria are primarily discernible within the IR channel, while the textural information in the visible spectrum is significantly diminished.

The observation reveals that the PCA method and several other comparative algorithms fail to effectively enhance the bacterial features, resulting in the poor visibility of the targets. In contrast, the SwinFusion model, the FusionGAN approach, and the proposed ITSF method achieve the favorable results by successfully capturing the infrared reflection from the IR channel. From the comprehensive perspective, the proposed model demonstrates the strongest contrast for the bacteria, ensuring the optimal visibility and the structural clarity of the bacterial targets against the low brightness within the visible spectrum.

### 4.5. Object Detection Performance Comparison

To further validate the efficacy of the proposed image fusion method, we utilize the state-of-the-art detection network, namely YOLOv11 [49], to quantify the object detection performance on both the source images and the fused results produced by various algorithms.

The bacterial targets were manually annotated using LabelImg by professional personnel. Each annotation was verified through rigorous comparison with the physical samples to ensure the accuracy of the ground truth. To maintain the experimental consistency, the data distribution for the fused images of each method is kept identical to that of the source images, with the dataset partitioned into the training, validation, and test sets at a ratio of 7:1:2.

Table 2 presents the performance metrics of the object detection results obtained from both the source images and the fused images produced by the comparative methods. Specifically, ${mAP}_{50}$ denotes the mean Average Precision (mAP) value at an Intersection over Union (IoU) threshold of 0.5, whereas ${mAP}_{50:90}$ represents the average mAP calculated across the IoU thresholds ranging from 0.5 to 0.95 with an increment of 0.05.

In this specific task, the primary emphasis is placed on the comprehensive detection rate of the bacteria, which is characterized by the Recall rate. As indicated in the experimental results, the proposed method achieves optimal performance in Recall, as well as in the ${mAP}_{50}$ and ${mAP}_{50:90}$ metrics. This superior performance demonstrates that the proposed ITSF successfully preserves the critical semantic information and the textural features necessary for the downstream computer vision tasks, effectively enhancing the visibility of the bacterial targets for the automated detection systems.

Moreover, we present the visual detection results corresponding to the different fusion methods to analyze the respective limitations in the object detection performance. As shown in the figures, the proposed method demonstrates the favorable performance both on the surface of the tissue culture and in the surrounding regions.

**Figure 8:**
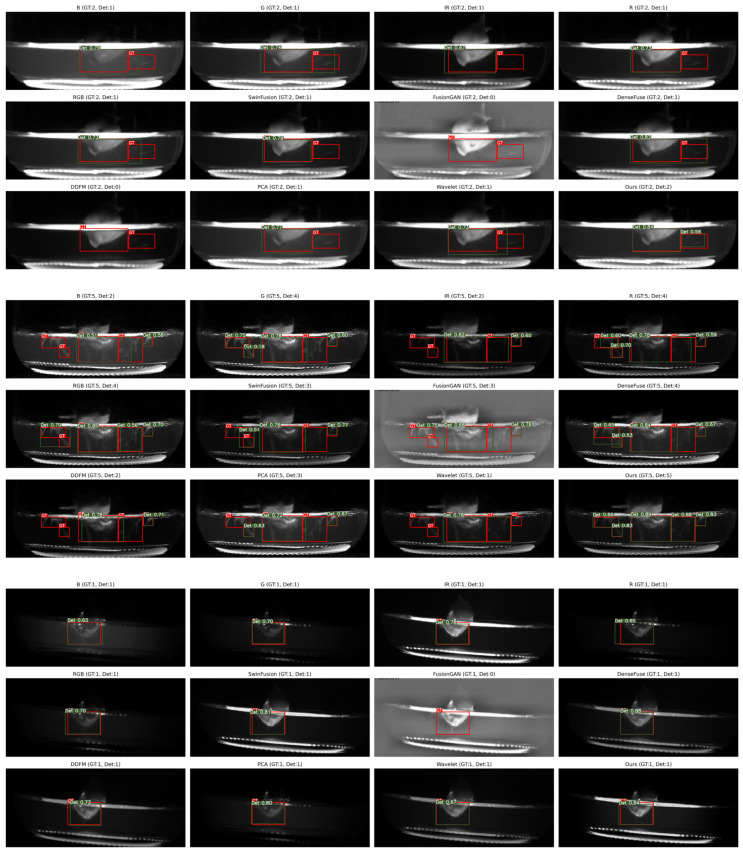
Visualization of object detection performance under YOLOv11 using different image fusion methods.

More precisely, the proposed ITSF achieves an mAP_50_ of 0.949, realizing the high-precision detection of the bacteria. As shown in Figure 8, the detection efficacy is particularly significant for the bacterial targets located in the peripheral regions far from the tissue culture. This indicates that our method effectively enhances the target features and suppresses the background interference, thereby facilitating the high Recall and the robust performance required for the automated monitoring of the biological samples. Notably, in scenarios where visible light quality is compromised (e.g., low-intensity conditions), the ITSF model successfully leverages the complementary synergy between visible and infrared modalities, significantly outperforming traditional RGB-based detection.

### 4.6. Ablation Study

The performance of the proposed model relies on the meticulously designed network architecture and the loss functions.

On one hand, the texture domain and the intensity domain perform the shallow feature processing for the visible and infrared modalities, respectively. This design aims to enhance the textural information of the visible images and the intensity information of the infrared image. Subsequently, the RSTB is employed for the deep feature extraction to capture the global context from the shallow features. Furthermore, the RCSTB facilitates the comprehensive integration of complementary information and the long-range dependencies across both the intra-domain and the inter-domain. This architecture enables the network to perceive global intensity from a holistic perspective, achieving the robust saliency perception of the bacterial targets. To evaluate the structural necessity of the proposed architecture, we performed an ablation study on the key feature modules while maintaining the optimized loss function. This setup ensures that the performance gains can be directly attributed to the modules’ ability to capture complex bacterial textures under an ideal supervisory signal.

On the other hand, entropy weighted SSIM loss, texture loss, and intensity aware dynamic gain guided loss drive the model to achieve the effective structural preservation, the texture detail retention, and the appropriate intensity control. To quantify the performance gains of these proposed loss functions over generic alternatives [33], we conducted a series of ablation studies while maintaining constant model architecture.

#### 4.6.1. TEM

Current visible and near-infrared image fusion methods [26] typically transform the RGB images into the YCbCr color space to extract the Y channel for the fusion process. The luminance component Y is generally calculated as follows:

(22) Y=0.299×R+0.587×G+0.114×B.

In fact, the chrominance channels (Cb and Cr) provide essential representative information regarding the imaging scene. However, the majority of the existing visible and near-infrared image fusion frameworks either neglect these channels or perform rudimentary fusion, leading to significant information loss.

To address this, the proposed TEM differs from the conventional approaches by utilizing the three RGB channels as direct inputs. By employing multi-scale convolutional operations followed by concatenation, the module not only preserves the information across all three channels but also maximizes the retention of the image textural details from a multi-scale perspective.

**Figure 9:**
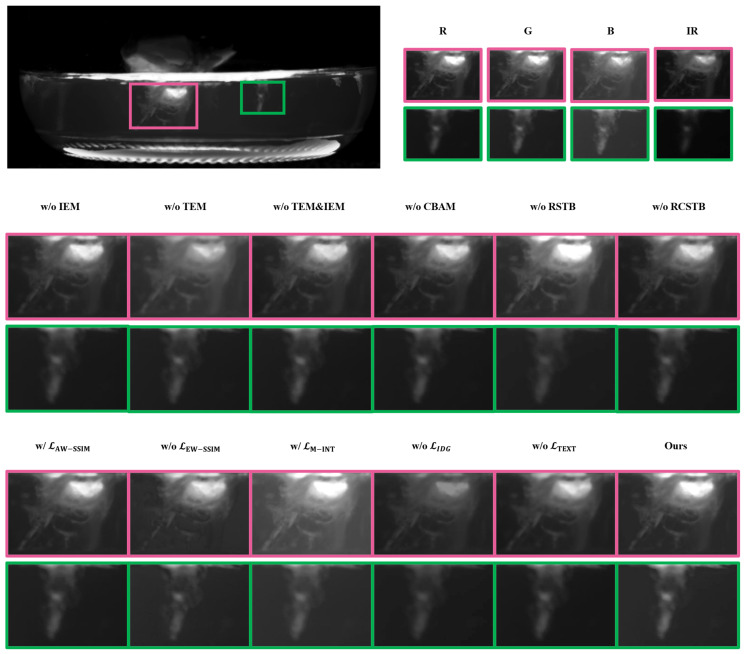
The results of the ablation experiments for the different modules and the loss functions. The visual comparisons illustrate the individual contributions of specific components to fusion performance, particularly in the scenarios where the bacteria are conspicuous in the visible (RGB) channels.

In the ablation study, we substituted the TEM with three standard convolutional layers to evaluate its specific contribution. As illustrated in Figure 9, in the absence of the TEM, the textural features of the bacteria cannot be effectively preserved. Additionally, the fused image suffers from the increased background noise, which obscures the prominence of the bacteria. This result underscores the critical function of the module in maintaining the textural richness and suppressing the background noise.

#### 4.6.2. IEM

Within the infrared and visible light image fusion task, the infrared image carries the infrared reflection information of the scene, which serves as the critical foundation for detecting the bacterial colonies.

**Figure 10:**
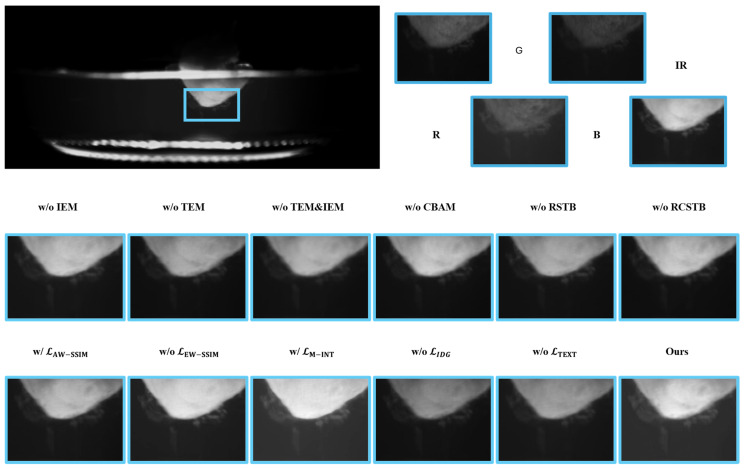
The results of the ablation experiments for the different modules and the loss functions. The visual comparisons illustrate the individual contributions of specific components to fusion performance, particularly in the scenarios where the bacteria are conspicuous in the IR channels.

In the ablation study, we replaced the IEM with three standard convolutional layers to perform the feature extraction. It is noteworthy that the ablation of the IEM leads to a marginal increase in SD and SCD, as shown in Table 3. However, this localized numerical gain does not translate to superior fusion quality. While higher SD and SCD values might suggest superior visual contrast and information retention in generic fusion tasks, they do not necessarily translate to enhanced bacterial saliency. Crucially, the proposed ITSF outperforms the ablation variants across the other four key fusion metrics and achieves the highest ${mAP}_{50}$ and ${mAP}_{50:95}$. This underscores that the inclusion of the IEM ensures a more focused enhancement of target-related saliency, prioritizing downstream detection performance over global statistical consistency. As illustrated in Figure 10, when the bacterial features within the IR modality are more prominent among the source four channel images, the absence of the intensity module leads to the loss of the specific bacterial details.

Moreover, the fused results without the IEM exhibit diminished luminance, making the targets less distinguishable within the image. These observations demonstrate that the IEM plays an irreplaceable role in the preservation and the enhancement of the critical infrared intensity information, ensuring that the signatures of the bacteria are effectively maintained.

#### 4.6.3. CBAM

The CBAM is designed to integrate the channel and spatial attention mechanisms, assisting the model in filtering the most critical information while eliminating the redundant features. This is particularly vital for the texture information module, where the multi-scale convolutional operations followed by the concatenation generate high channel information redundancy.

Specifically, the convolution and repeated concatenation of the three visible channels result in an excessive amount of overlapping data. In the spatial domain, the information extracted by the convolutional kernels of different sizes also exhibits significant duplication. Consequently, the CBAM is essential to preserve the pivotal channel and spatial features, thereby reducing the model complexity, enhancing the computational efficiency, and preventing the overfitting.

In the ablation study, when the CBAM was removed, the model achieved suboptimal performance across several objective metrics as shown in Table 3. However, the object detection metrics indicated that the performance was less than ideal. Furthermore, as illustrated in the images of Figure 9 and Figure 10, in the absence of the CBAM, certain bacterial features were not effectively enhanced, making it difficult for the human eyes to distinguish them clearly. This underscores the importance of the attention mechanism in prioritizing the salient biological targets within the complex fusion scenarios.

#### 4.6.4. RSTB

In the deep learning-based image fusion frameworks, effectively modeling and integrating the global semantic dependencies and the long-range contextual information remains a pivotal challenge for enhancing the fusion quality. Traditional convolutional neural networks are constrained by the inherent limitations of the local receptive field, making it difficult to establish direct associations between distant pixels or regions. This often results in the poor holistic perception of the image.

The RSTB, built upon the Swin Transformer framework, enables the model to transcend the local window constraints. By facilitating the cross-window information interaction, the RSTB enhances the global perception of the entire image. This mechanism improves the textural details in the dark regions and regulates the overall luminance to prevent the local overexposure or the underexposure, thereby maintaining the visual naturalness and the coordination of the fused results [34].

In the ablation study, we removed the RSTB from both the feature extraction and the image reconstruction stages. As illustrated in Figure 10, in the absence of the RSTB module, the bacterial targets across different regions exhibit significant brightness disparities. This lack of the global luminance regulation leads to inconsistent visual quality. These observations confirm that the RSTB is essential for achieving the balanced intensity distribution and the superior structural integrity in the complex imaging scenarios.

#### 4.6.5. RCSTB

The RCSTB comprises the intra domain fusion unit based on the self-attention mechanism and the inter-domain fusion unit based on the cross-attention mechanism. This architecture is designed to fully aggregate the long-range dependencies and the global interactions both within and across the source images.

In particular, the intra domain fusion unit aggregates the long-range spatial dependencies within the visible or infrared images through the self-attention mechanism, thereby effectively capturing the global contextual structure within a single modality. Conversely, the inter-domain fusion unit utilizes the cross-attention mechanism to establish the deep interaction between the visible and infrared modalities. This allows the features from different domains to guide each other, achieving the complementary enhancement of both the bright and dark regions.

As illustrated in the ablation results of Figure 9, upon the removal of the RCSTB, the fusion model fails to effectively regulate the intensity of the fused image. This leads to an overall darkening of the bacteria, which significantly hinders the visibility of the targets. These observations confirm that the CRSTB is essential for maintaining the optimal luminance and ensuring the effective integration of multimodal information.

#### 4.6.6. Analysis of Entropy Weighted SSIM Loss

The inclusion of the SSIM loss is primarily intended to facilitate the consistency in the structural similarity between the fused results and the source images, ensuring that the output maintains high structural fidelity relative to the input while avoiding the structural distortion or the artifacts. In conventional visible and near-infrared image fusion tasks, the average weighting method is typically employed to balance the SSIM similarity between the fused image and the two source images. However, in our specific task involving the four channel source images, we adopt the information entropy weighting strategy to align the fused results more closely with the source images that contain the higher information content.

To validate the performance of $L_{EW-SSIM}$, we conducted the ablation experiments comparing our approach against the average weighted SSIM loss ($L_{AW-SSIM}$) and a configuration without the SSIM loss. As indicated in Table 3, when the SSIM loss is absent, the SCD metric drops to the lowest value among all the ablation configurations, suggesting that the model fails to effectively balance the complementary information across the various images.

While the situation improves with the implementation of $L_{AW-SSIM}$, the performance regarding the object detection metrics remains relatively low. Furthermore, the visual evidence presented in Figure 9 and Figure 10 demonstrates that both the baseline configurations result in an inadequate integration of the information from the four source channels. Consequently, these methods fail to effectively accentuate the bacterial features. These findings confirm that entropy weighted SSIM loss is essential for maintaining structural integrity and enhancing the saliency of the bacterial targets.

#### 4.6.7. Analysis of Intensity Aware Dynamic Gain Guided Loss

The inclusion of the intensity loss is primarily intended to enhance the luminance of the desired targets, thereby improving the discernibility of the bacterial colonies. In conventional frameworks, the visible and near-infrared image fusion typically adopts the maximum intensity principle. This approach ensures that the complementary brightness of the targets is preserved, such as the thermal targets in the infrared image and the texture information in the visible image.

However, relying solely on this principle is insufficient for the tissue culture dataset. Due to the light scattering within the culture medium, the background noise in certain channels exhibits excessively high luminance. Simple fusion based on maximum intensity results in the loss of the rich textural information of the bacteria. Consequently, this traditional strategy is suboptimal for selecting the most informative complementary features in complex biological scenarios.

To evaluate the performance, we conducted the ablation experiments comparing $L_{IDG}$ against the maximum intensity loss $L_{M-INT}$ and a configuration without the intensity loss. As illustrated in Figure 9 and Figure 10, when the maximum intensity loss is applied, the fused images exhibit significant overexposure artifacts. Conversely, in the absence of intensity loss, the images appear dim and lack the necessary contrast. In both cases, the bacterial targets fail to be prominently highlighted. These results confirm that the proposed intensity loss effectively balances the target enhancement and the background suppression, ensuring the optimal visibility of the bacteria.

#### 4.6.8. Analysis of Texture Loss

The primary objective of incorporating the texture loss is to preserve the intricate textural details within the fused images, which is essential for the bacteria detection task. To evaluate its significance, we conducted the ablation study as presented in Table 3. In the absence of texture loss, besides the decline in several objective metrics, the object detection performance suffers the most significant degradation. Specifically, the ${mAP}_{50}$ and ${mAP}_{50:95}\;$ reach the lowest value among all the ablation configurations.

Moreover, the visual results in Figure 9 and Figure 10 demonstrate that the image sharpness is noticeably compromised when the gradient loss is omitted. The bacterial boundaries appear blurred, which negatively impacts the discernibility of the targets. These findings confirm that texture loss plays a vital role in maintaining structural clarity and providing the high-quality information required for the downstream automated detection systems.

## 5. Discussion

The ITSF model developed in this study achieved a mAP_50_ of 0.949 in detecting bacterial contamination in *Alocasia* explants. This study addresses a critical technological gap in the automated detection of plant tissue culture contamination. Currently, large-scale micropropagation still heavily relies on manual visual inspection, which is not only labor-intensive but also highly subjective, particularly in identifying early-stage contamination during the translucent phase. Within plant tissue culture environments, bacteria typically manifest as translucent biofilms or filaments, rendering them highly susceptible to confusion with medium scattering or complex surface textures of explants under single spectral channels. Experiments indicate that images from all four channels may contain unique bacterial information. Specifically, the three visible light channels capture bacterial texture information, while the infrared channel captures bacterial infrared reflectance information, simultaneously reducing background scattering noise and interference from tissue culture surface textures.

The ITSF model is an improved version based on SwinFusion. In the feature extraction part, it implements multi-scale texture extraction for the visible light channel. Compared to SwinFusion’s strategy of directly using the Y channel from RGB images converted to YCrCb color space for fusion, this enhancement significantly improves the extraction of bacterial texture features and better preserves effective information from the three RGB channels. For the infrared channel, the model employs DenseNet for intensity feature extraction, which retains the characteristic information of the original infrared images more effectively compared to traditional multi-layer convolutional structures. Furthermore, the CBAM attention mechanism is incorporated to further integrate the features extracted from the two branches and filter out redundant information. In terms of computational efficiency, the proposed ITSF-YOLO framework achieves a total inference time of 2.89 s for a high-resolution (2000×870 pixels) image on an NVIDIA RTX 4090 GPU.

Compared to SwinFusion and conventional deep learning-based image fusion models, the ITSF model incorporates targeted improvements in its loss functions. The Entropy Weighted SSIM Loss enables the model to dynamically learn from spectral images with richer information, ensuring that the fused image contains more comprehensive and detailed content. The intensity aware dynamic gain guided loss offers a novel approach for enhancing target salience. Commonly used maximum intensity loss often leads the model to favor the brightest regions across images. When overexposed areas in certain images obscure target information from others, this loss may cause the fused output to emphasize overexposed regions rather than the actual targets. In contrast, the intensity aware dynamic gain guided loss not only highlights texture rich targets but also dynamically adjusts image intensity by suppressing overexposed areas while enhancing brightness in regions with rich texture, thereby effectively improving target saliency.

From a technical perspective, this work still has room for improvement. First, the study is limited in terms of sample diversity, as it primarily uses *Alocasia* Explants and does not yet cover other micropropagation species or different types of bacterial contamination. Future research could include a wider variety of contaminated culture samples to validate the generalization ability of the algorithm, thereby advancing toward a universal automated recognition system for micropropagation. Second, the study’s current scope is primarily focused on standardized Alocasia explants, specifically using sliced stem and bud tips. In our experiments, bacterial contamination typically manifests within a 2–3 day window post-culture. As plant tissues develop, physiological changes may alter the spectral background, potentially complicating the detection task. Future research will involve collecting cross-batch data from different industrial production cycles and diverse micropropagation species to enhance the model’s generalization and move toward a more universal recognition system. Third, from the perspective of equipment cost, adopting LEDs with specific wavelength ranges [50] to replace expensive multispectral filters can significantly reduce the overall cost and achieve higher efficiency. Meanwhile, the ITSF algorithm currently requires substantial computational resources for both training and inference, and the image fusion process remains time-consuming. In the future, the algorithm could be optimized through improved design or techniques such as model pruning and quantization to enable lightweight deployment, which would reduce the cost of automation equipment implementation.

## 6. Conclusions and Outlook

In this paper, we addressed the critical task of bacterial detection within the plant tissue culture, utilizing *Alocasia* Explants as the primary subjects. We have established a multispectral image acquisition system, constructed a tissue culture dataset for visible-infrared multispectral imagery, and developed the ITSF algorithm to achieve efficient image fusion. The proposed ITSF architecture utilizes the TEM to extract the textural details from the visible spectral channels and the IEM to capture the intensity information from the infrared channel, thereby fully mining the informative features within the source images. Subsequently, the RSTB and the RCSTB architectures regulate the overall brightness from a global perspective, effectively enhancing the contrast between the bacteria and the background. Finally, the composite loss function is introduced to achieve the maximum extraction of the image information, further improving the saliency of the bacteria.

The experimental results demonstrate that the proposed method provides both the superior visual perception and the outstanding objective metrics. Notably, the system achieves a high-precision detection performance of 0.949 in terms of the ${mAP}_{50}$, which represents the highest value among the mainstream image fusion architectures. This performance successfully satisfies the industrial application standards for the high-precision inspection. By providing the early and the accurate identification of the bacterial threats, the proposed system contributes to the reduction of the economic losses in the large-scale micropropagation facilities, thereby enhancing the overall sustainability of the plant biotechnology industry.

## Figures and Tables

**Table 1:** Average objective evaluation metrics of different fusion methods on our dataset. The best and second-best performances are highlighted in bold red and bold blue, respectively. The arrow ↑ denotes that higher values is preferred.

Metric	SD ↑	SCD ↑	Qabf ↑	PSNR ↑	FMI ↑	VIF ↑
DDFM	52.764	−0.617	0.073	14.841	0.566	0.249
DenseFuse	51.886	−0.074	0.411	22.913	**0.761**	0.799
FusionGAN	38.157	−1.102	0.241	9.299	0.687	0.780
PCA	**55.742**	**0.218**	**0.462**	**24.953**	0.742	0.837
SwinFusion	55.510	0.024	0.417	21.932	0.746	**1.224**
Wavelet	52.281	−0.072	0.378	22.646	0.717	0.948
Ours	**60.650**	**0.496**	**0.437**	**23.174**	**0.760**	**1.415**

**Table 2:** Object Detection Metrics Display. The best and second-best performances are highlighted in bold red and bold blue, respectively. The arrow ↑ denotes that higher values is preferred.

Metric	Precision ↑	Recall ↑	${\mathrm{mAP}}_{50}\;↑$	${\mathrm{mAP}}_{50:95}\;↑$
R	**0.978**	0.830	0.910	0.670
G	0.957	0.830	0.910	0.664
B	0.976	0.774	0.884	0.618
IR	0.913	0.792	0.884	0.638
RGB	0.902	0.868	0.921	0.675
DDFM	0.933	0.792	0.884	0.607
DenseFuse	1.000	0.809	0.909	0.667
FusionGAN	0.927	0.736	0.851	0.608
Wavelet	**1.000**	0.788	0.894	0.662
PCA	0.92	0.827	0.916	0.651
SwinFusion	0.9	**0.904**	**0.941**	**0.678**
Ours	0.972	**0.906**	**0.949**	**0.712**

**Table 3:** Presentation of Ablation Experiment Indicators. The best and second-best performances are highlighted in bold red and bold blue, respectively. The arrow ↑ denotes that higher values is preferred.

Methods	SD↑	SCD↑	Qabf↑	PSNR↑	FMI↑	VIF↑	${mAP}_{50}↑$	${mAP}_{50:95}↑$
w/o IEM	**63.002**	**0.587**	0.397	21.934	**0.759**	1.413	0.91	0.633
w/o TEM	61.267	0.287	0.427	19.493	0.756	1.294	0.925	0.686
w/o TEM & IEM	59.294	0.333	0.428	22.842	0.756	1.372	0.884	0.631
w/o CBAM	**62.422**	**0.562**	0.396	21.877	**0.759**	1.398	0.893	0.677
w/o RSTB	60.076	0.42	0.427	22.967	0.756	1.367	0.918	0.667
w/o RCSTB	58.291	0.204	0.424	22.812	0.758	1.358	0.93	0.699
w/$L_{AW-SSIM}$	60.923	0.509	0.434	23.056	0.758	1.407	0.884	0.618
w/o $L_{EW-SSIM}$	58.306	0.196	0.398	22.857	0.741	1.367	0.917	0.676
w/$L_{M-INT}$	60.429	0.448	0.432	23.056	0.738	1.407	0.915	0.645
w/o $L_{IDG}$	60.877	0.389	0.424	22.798	0.755	**1.553**	**0.934**	**0.7**
w/o $L_{TEXT}$	59.626	0.407	**0.435**	**23.133**	0.753	1.314	0.862	0.61
Ours	60.65	0.496	**0.437**	**23.174**	**0.760**	**1.415**	**0.949**	**0.712**

## Data Availability

The data and the conclusions of this study are available from the corresponding: author upon reasonable request.

## Abbreviations

The following abbreviations are used in this manuscript:

TEM	Texture Extraction Module
IEM	Intensity Extraction Module
CBAM	Convolutional Block Attention Module
RSTB	Residual Swin Transformer Block
RCSTB	Residual Cross-Swin Transformer Block
CRB	Convolutional Reconstruction Block
BN	Batch Normalization
LReLU	Leaky Rectified Linear Unit
DC	Dense Convolution
STL	Swin Transformer Layer
MLP	Multi-Layer Perceptron
MSA	Multi-head Self-Attention
MCA	Multi-head Cross-Attention
GELU	Gaussian Error Linear Unit
CSTL	Cross-Swin Transformer Layer
SD	Standard Deviation
SCD	Sum of Correlation Differences
VIF	Visual Information Fidelity
PSNR	Peak Signal-to-Noise Ratio
FMI	Feature Mutual Information
IDG	Intensity aware Dynamic Gain
